# Exploring Mitochondrial Interactions with Pulsed Electromagnetic Fields: An Insightful Inquiry into Strategies for Addressing Neuroinflammation and Oxidative Stress in Diabetic Neuropathy

**DOI:** 10.3390/ijms25147783

**Published:** 2024-07-16

**Authors:** Diego Chianese, Massimo Bonora, Maria Sambataro, Luisa Sambato, Luca Dalla Paola, Elena Tremoli, Ilenia Pia Cappucci, Marco Scatto, Paolo Pinton, Massimo Picari, Letizia Ferroni, Barbara Zavan

**Affiliations:** 1 Medical Sciences Department, University of Ferrara, 44133 Ferrara, Italy; diego.chianese@unife.it (D.C.); massimo.bonora@unife.it (M.B.); paolo.pinton@unife.it (P.P.); 2 Endocrine, Metabolism and Nutrition Disease Unit, Ca’ Foncello Sant Mary Hospital, 30193 Treviso, Italysambado@unitv.it (L.S.); 3 Maria Cecilia Hospital, GVM Care & Research, Cotignola, 48033 Ravenna, Italy; dallapaolaluca@unife.it (L.D.P.); etremoli@unife.it (E.T.); ileniacpucci@unife.it (I.P.C.); 4 Department of Economics, Science, Engineering and Design, San Marino University, 47890 Città di San Marino, San Marino; scatto@studio.unismar.sm; 5 Translational Medicine Department, University of Ferrara, 44133 Ferrara, Italy; massimo.picari@unife.it

**Keywords:** pulsed electromagnetic fields, diabetic foot, reactive oxygen species, cytokines, wound healing, complex magnetic fields

## Abstract

Pulsed electromagnetic fields (PEMFs) are recognized for their potential in regenerative medicine, offering a non-invasive avenue for tissue rejuvenation. While prior research has mainly focused on their effects on bone and dermo-epidermal tissues, the impact of PEMFs on nervous tissue, particularly in the context of neuropathy associated with the diabetic foot, remains relatively unexplored. Addressing this gap, our preliminary in vitro study investigates the effects of complex magnetic fields (CMFs) on glial-like cells derived from mesenchymal cell differentiation, serving as a model for neuropathy of the diabetic foot. Through assessments of cellular proliferation, hemocompatibility, mutagenicity, and mitochondrial membrane potential, we have established the safety profile of the system. Furthermore, the analysis of microRNAs (miRNAs) suggests that CMFs may exert beneficial effects on cell cycle regulation, as evidenced by the upregulation of the miRNAs within the 121, 127, and 142 families, which are known to be associated with mitochondrial function and cell cycle control. This exploration holds promise for potential applications in mitigating neuropathic complications in diabetic foot conditions.

## 1. Introduction

Diabetes, marked by chronic hyperglycemia, is a global crisis, with the diabetic population projected to reach 592 million people by 2035. Diabetic neuropathy (DN), impacting 50–60% of patients, entails progressive nerve damage, leading to atrophied nerves and irreversible impairment, clinically expressed through sensations ranging from numbness to persistent pain [1]. Investigating the intricate interplay between mitochondrial function and pulsed electromagnetic fields (PEMFs) holds potential for understanding neuroinflammation and oxidative stress in DN.

Despite hyperglycemia being a recognized DN driver, its intricate mechanisms remain incompletely understood. Nerve cells, reliant on external glucose concentrations, are susceptible to hyperglycemic injury [2]. Dysregulated glucose levels, showing a 4–5-fold elevation in individuals with diabetes, are linked to reduced neurotrophic support, causing neuronal dysfunction. Addressing DN-associated pain is challenging due to inadequate diagnostic criteria and limited treatment options. The pharmacological arsenal includes tricyclic antidepressants, selective serotonin, noradrenaline reuptake inhibitors, anticonvulsants, and opioids. However, only duloxetine and pregabalin are Food and Drug Administration (FDA)-approved, with limitations in efficacy, cost, and adverse effects [3].

Diabetic peripheral neuropathy results in pain and sensory loss, impacting people’s quality of life. Hyperglycemia initiates pathways like polyol, protein kinase C (PKC), and mitogen-activated protein K (MAPK) signaling, along with advanced glycation end-product (AGE) assembly, leading to the generation of inflammatory mediators. AGE accumulation triggers the nuclear factor kappa-light-chain-enhancer of the activated B cell (NF-κB)-mediated release of cytokines and chemokines. The receptor for advanced glycation end-product (RAGE) activation induces the NF-κB cascade, causing neuronal apoptosis and suppressing antioxidant genes via nuclear factor erythroid 2-related factor 2 (Nrf2) downregulation.

Hyperglycemia-induced inflammation affects neuronal structure, leading to myelin glycosylation, immune cell infiltration, and cytokine release, creating a positive feedback loop which worsens nerve damage. Cytokines sensitize receptors, causing neuropathic pain [4]. Neuroinflammation, via MAPK and tumor necrosis factor (TNF)-α, damages the nerves, reducing blood perfusion and neurotrophic support. The released chemokines induce hyperalgesia, while diabetes-induced hypoxia worsens inflammation via inducible nitric oxide synthase (iNOS), releasing nitric oxide (NO). Inflammatory cascades, cytokine upregulation, and neuroimmune pathways contribute significantly to peripheral nerve damage in diabetic neuropathy. In summary, hyperglycemia triggers oxidative stress and inflammation, intertwining and complicating the damage to neurons [5].

Mitochondrial electron transport chain activity rises, generating superoxide anions. This excess superoxide production leads to the formation of other types of reactive oxygen species (ROS) and peroxynitrite, causing structural and functional damage. Peroxynitrite-induced DNA damage activates poly (ADP-ribose) polymerase (PARP), depleting NADH and driving cells into necrosis, releasing cellular debris, and triggering local inflammation [6]. The interplay of superoxides and NO results in the formation of peroxynitrite, intensifying the damage. Hyperglycemia-induced oxidative stress also activates cellular pathways such as Nrf2 and NF-*κ*B. Nrf2 activation enhances antioxidant and cytoprotective enzyme production, providing cellular defense against oxidative stress [6].

However, persistent Nrf2 activation is hampered by hyperglycemia-mediated extracellular signal-regulated kinase (ERK) activation, leading to failed redox homeostasis in a diabetic state. Oxidative stress-induced inflammation stimulates the NF-*κ*B, activator protein 1 (AP-1), and MAPK pathways. ROS trigger IKK activation, phosphorylating IkappaB kinase (I*κ*B) for ubiquitin-mediated proteasomal degradation, freeing NF-*κ*B to translocate into the nucleus and induce the transcription of inflammatory cytokines [7]. Upstream, p38 MAPK activation occurs through hyperglycemia-mediated apoptosis signal-regulating kinase 1 (ASK1) or, indirectly, through oxidative stress. Stress-activated protein kinases such as c-Jun NH2-terminal kinases (JNKs) further activate AP-1, contributing to collagenase, transforming growth factor (TGF)-1β, and cytokine production. Nrf2-NF-*κ*B crosstalk is pivotal, with Nrf2 inhibiting NF-*κ*B activation, thereby maintaining cellular homeostasis.

This delicate balance is disrupted in diseases with excessive oxidative stress, leading to detrimental consequences. In peripheral nerve damage and neuroinflammation, neuroglial cells act as a connecting link between oxidative stress and inflammation [8]. Oxidative damage to glia results in proinflammatory cytokine release, activating neuronal cell membrane receptors and initiating inflammatory pathways. Evidence also supports the role of vascular inflammation in diabetic neuropathy, indicating that neuroinflammation, inflammation, and oxidative stress collectively underlie peripheral nerve damage in vasa nervorum and neuroglial cells [9]. PEMFs are a scientifically validated tool in modern medicine, with diverse applications, positively influencing cellular behavior for tissue regeneration, inflammation reduction, and modulation of the peripheral nervous system [10]. Different treatment programs varying in frequency, intensity, and duration have been developed and validated for PEMFs [10].

Ongoing research explores the intricate mechanisms behind the processes related to inflammation. Key findings include the induction of cellular transcription, the regulatory effects on lymphocyte proliferation, and the potential involvement of Ca^2+^ channel activation. Magnetic fields may influence voltage-dependent calcium channels through the Lorentz force, enhancing their permeability [11]. Resonance dependence on frequency, particularly cyclotron resonance, and the role of solitons in cellular processes such as ATP hydrolysis are highlighted [11]. In recent years, great attention has been placed on the action of such a technology, in particular in the treatment of diseases related to the central and peripheral nervous systems [12]. Preliminary in vitro results have confirmed the positive effects of PEMFs on glial tumor cell lines [13]. Ultra-short pulsed electric fields of a high amplitude (from a few to hundreds of kV/m) and short duration (from a few milliseconds to some microseconds) have emerged as a new physical agent, which can facilitate temporary blood–brain barrier disruption, thus allowing for the easy and effective penetration of therapeutic reagents in glioblastoma [13]. In particular, a specific signal of this class, the so-called PEF-5 (i.e., five electric pulses lasting 40 µs, pulse amplitude 0.3 MV/m, repeated at 1 Hz), irreversibly affect medulloblastoma (MB) cells [13]. In the study, exposure to PEMFs substantially influenced medulloblastoma cells by regulating many genes involved in hypoxia, inflammation, and p53/cell cycle checkpoints, resulting in a reduction in cell capacity to form new neurospheres and also transmigrate in vitro [14].

To this end, we have studied the biological effects of PEMFs in light of their different biological applications, as demonstrated by publications directed at confirming not only their biological safety on cellular systems such as mesenchymal stem cells (MSCs) but also their direct contribution to the healing process of skin and bone wounds [15,16,17,18]. Our research has demonstrated the impact of PEMFs on MSCs, fostering their differentiation into osteoblasts and vascular cells crucial for bone regeneration [16]. Additionally, PEMFs aid in managing chronic inflammation during the healing of challenging wounds such as those observed in diabetic foot ulcers [17,18,19]. Recently, our attention was focused on complex magnetic fields (CMFs), i.e., multi-frequency electromagnetic fields which vary in frequency and wave shape, allowing them to interact with specific cellular functions by means of dedicated programs [7].

We therefore decided to test two of them—the antioxidant support program (AOSP) and the anti-inflammatory program (AIP)—on glial cells derived from the in vitro differentiation of MSCs (i.e., non-tumor central nervous system cells), to study their effects on mitochondrial activity and miRNA expression.

## 2. Results

### 2.1. CMFs Do Not Affect the Biologically Related Proliferation Rate of Cells

**Figure 1:**
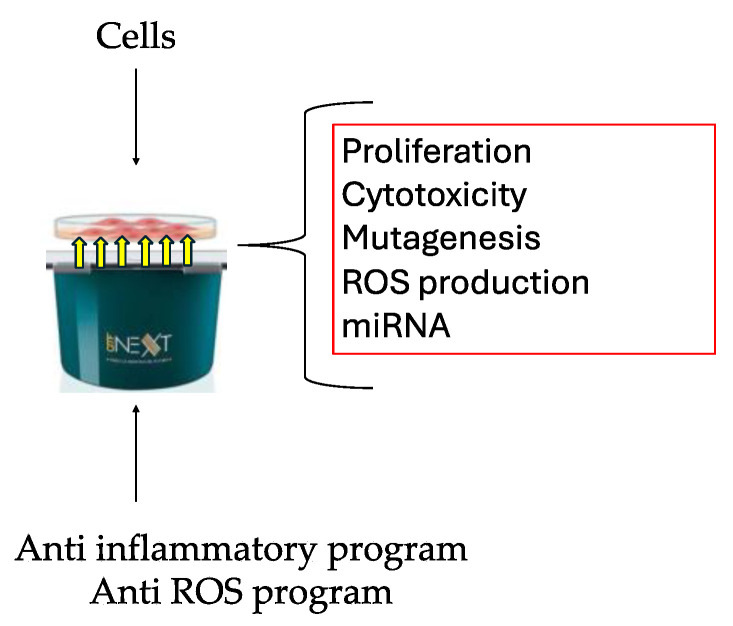
Experimental design sheet. Cells were treated with the anti-inflammatory or anti-ROS program and analyzed for proliferation my means of the MTT test, cytotoxicity with the hemolytic test, mutagenesis activity with the AMES test, and ROS production and miRNA through sequencing of the miRNA sequence.

Cell cultures, representing a spectrum of cell types including undifferentiated MSCs, differentiated MSCs (comprising neuron-like and glial-like cells), tumor glial cells (U251), and tumor neuron cells (B6/D2 F1), underwent extensive treatment with CMFs (Figure 1).

**Figure 2:**
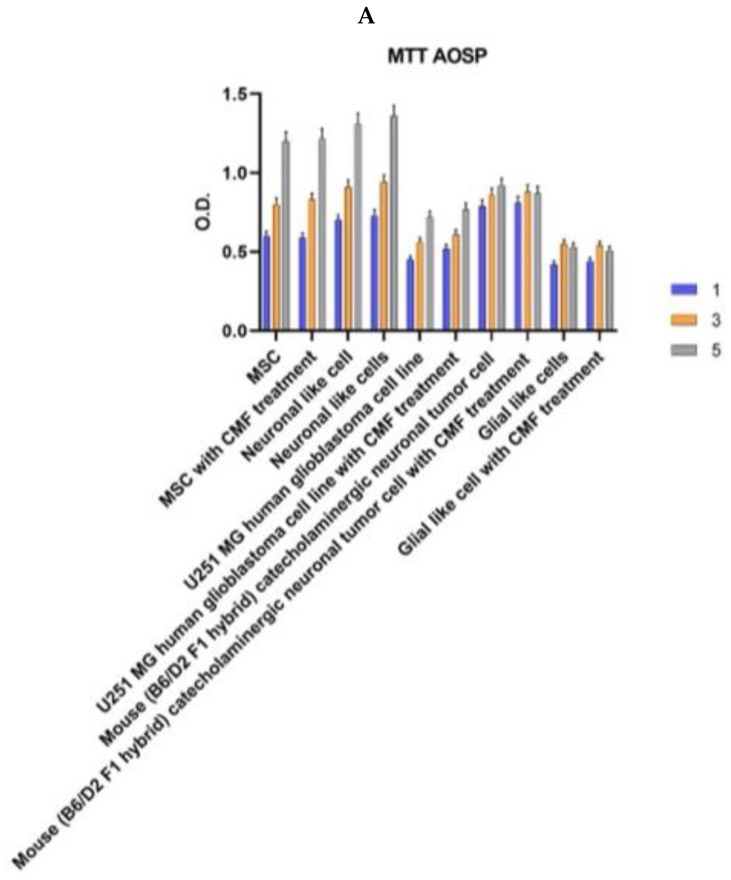

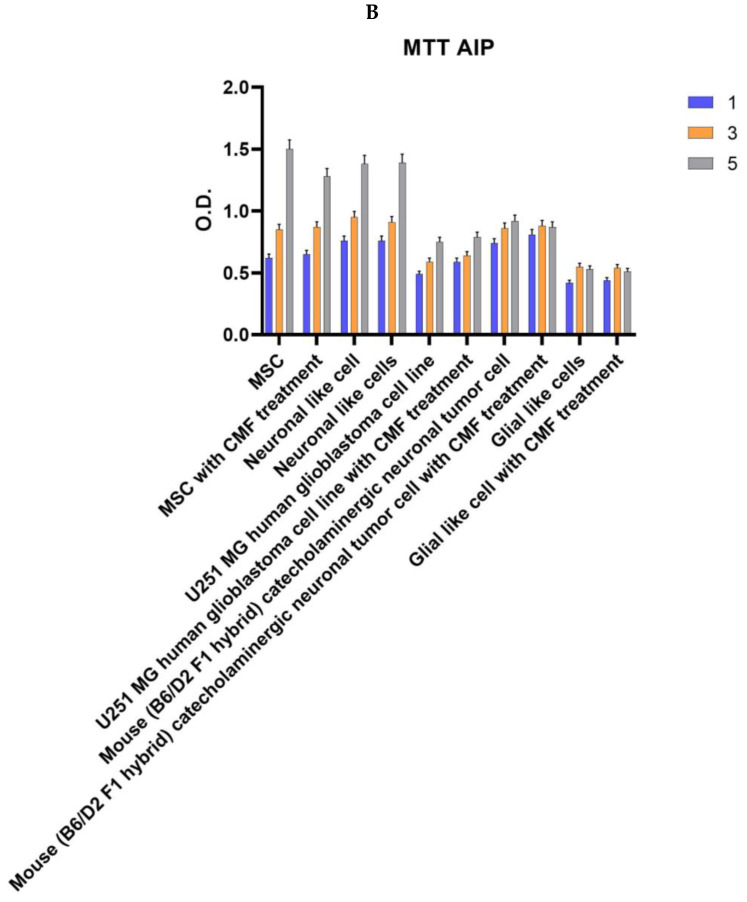
Cell proliferation of MSCs, neuronal-like cells, human glioblastoma-like cells, neuronal tumor cell line, and glial-like cells with or without CMF treatment. (**A**) Antioxidative stress program; (**B**) anti-inflammatory program. Tests were run after 1 (blue bars), 3 (orange bars), and 5 days (grey bars) of culturing and daily treatment. The graphs show the mean ± SD of three different experiments. O.D—optical density.

The assessment of cell line proliferation unfolded over the course of days 1, 3, and 5, employing the AOSP (Figure 2A) and the AIP to abate free radicals (Figure 2B). Impressively, neither program induced any discernible alteration in the normal proliferative processes associated with mitochondrial activity across all cell populations. This underscores the robustness and stability of cellular responses to the applied magnetic field interventions, suggesting a nuanced and well-preserved cellular homeostasis in the face of the CMF challenge.

### 2.2. CMFs Affect miRNA Synthesis Related to Anti-Inflammatory and Antioxidant Pathways

**Figure 3:**
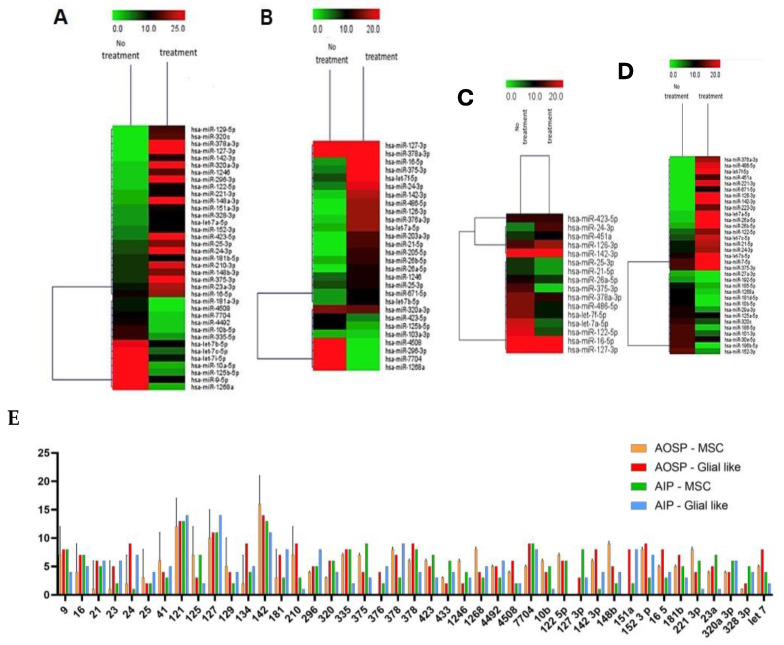
miRNA expression with sequencing (**A**–**D**) and droplet digital PCR (ddPCR) of the most important miRNAs analyzed: in yellow, glial-like cells treated with AIP; in gray, MSCs treated with AIP; in orange, glial-like cells treated with AOSP; and in blue, MSCs treated with AOSP. Levels obtained with no treatment are noted as 0 value.

**Figure 4:**
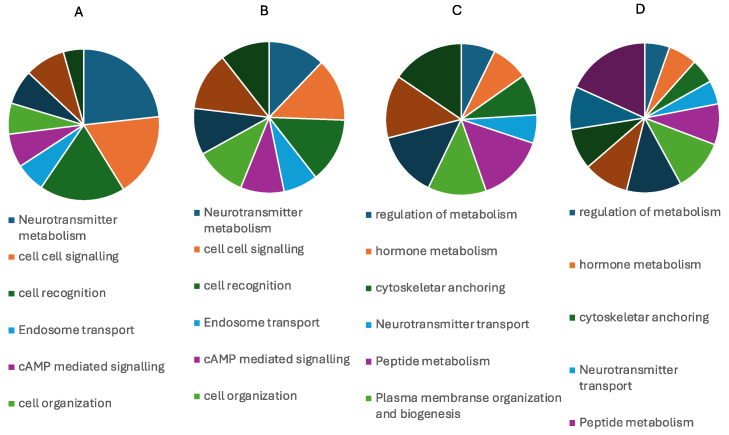
Pie chart related to the principal pathways of miRNA expression in (**A**–**C**) MSCs and (**B**–**D**) glial cells treated with AOSP (**A**,**B**) and AIP (**C**,**D**) compared to no treatment.

The profound effects induced by the AOSP and AIP treatments were examined concerning miRNA sequencing, both within MSC-derived glial-like cells and the control condition of MSCs. This type of analysis was performed by means of sequencing and bioinformatics analyses, with no statistical indication. An enrichment test was performed to give the direction of the pathways activated. Figure 3 thoughtfully encapsulates the intricate miRNA expression profiles, elegantly presented in the form of gene expression to confirm sequencing (Figure 3) and a pie graph to analyze the pathway. The figure also thoughtfully details the primary activating or suppressing pathways subject to regulation by miRNAs. In a nuanced breakdown, AOSP exhibited its regulatory prowess by orchestrating the expression of 37 miRNAs within the MSCs. These miRNAs, as vividly illustrated in Figure 4, demonstrated an upregulation of the processes pertaining to lipid transportation, secretory activity, and cellular organization. Simultaneously, there was a downregulation observed in the pathways associated with mitochondrial biogenesis and cell signaling. In a parallel narrative, AOSP exercised its influence over glial-like cells, governing the expression of 30 miRNAs (Figure 3A) able to orchestrate a complex interplay of molecular events and intricately modulate fundamental processes such as cell growth, vesicle-mediated transportation, and mitochondrial biogenesis. Indeed, the glial-like cells acted as pivotal regulators in fine-tuning the expression patterns of this diverse array of 30 microRNAs, as depicted in Figure 3B, which may harbor an intrinsic capability to influence antioxidant mechanisms within the cellular milieu. Such a modulation of antioxidant functions holds profound implications for cellular homeostasis and adaptive responses to oxidative stress, underscoring the multifaceted roles of these microRNAs in cellular physiology and pathology. On the other hand, AIP showcased its regulatory finesse by modulating the expression profiles of 16 miRNAs within MSCs (Figure 3C–E), which, in turn, orchestrated a multifaceted response, encompassing enhancements in plasma membrane organization, the facilitation of vesicle docking, and the promotion of cell proliferation, not only contributing to cellular homeostasis but also holding profound implications for MSC-mediated tissue repair and regeneration. This regulatory finesse not only underscored its significance in therapeutic interventions but also highlighted its pivotal role in orchestrating anti-inflammatory events within the cellular milieu. Furthermore, extending its regulatory prowess beyond MSCs and into the realm of glial-like cells, AIP exhibited its far-reaching impact by influencing the expression profile of 33 miRNAs (Figure 3B,D,E), fostering an increase in circadian clocking and plasma membrane organization. The microRNAs predominantly implicated in these physiological mechanisms belonged to three principal families—miR-121, miR-127, and miR-142—closely connected to the regulation of the pathways involved in extracellular matrix (ECM) restructuring and tissue repair, presenting a significant potential for the development of innovative and effective treatments for the management of neurodegenerative disorders (Figure 3E and Figure 4). As such, the exploration of the roles of miR-121, miR-127, and miR-142 not only advances our understanding of their biological relevance but also expands the horizon for targeted treatment development, potentially transforming the therapeutic landscape for neurodegenerative conditions. This extensive regulatory network further emphasizes the potential of AIP in modulating anti-inflammatory processes, holding promise for the development of novel therapeutic strategies aimed at mitigating inflammatory responses and the associated pathological conditions. This thorough investigation offers an intricate understanding of the multifaceted regulatory environment shaped by AOSP and AIP treatments, elucidating their distinct effects on a molecular scale. The evaluation of mitochondrial potential involved a detailed analysis of the mitochondria within MSC-derived glial-like cells, utilizing a nuanced morphological assessment.

### 2.3. CMFs Affect Mitochondrial Activity

**Figure 5:**
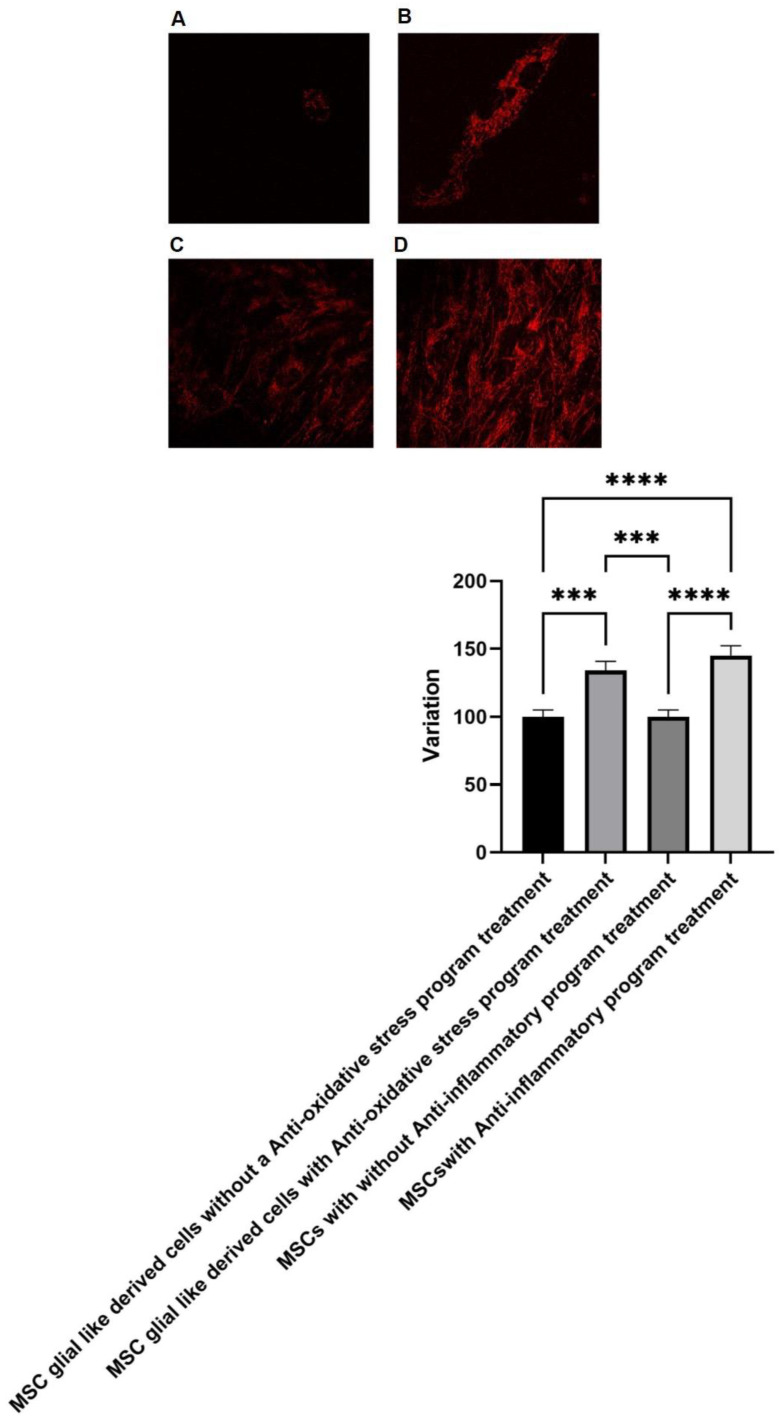
Mitochondrial membrane potential. (**A**) Representative images of MSC-derived glial-like cells without and (**B**) with antioxidative stress program treatment. (**C**) Representative images of MSCs without and (**D**) with anti-inflammatory program treatment. The absence of treatment is our negative control (*** *p* value ≤ 0.01, **** *p* value ≤ 0.005).

The comprehensive assessment of the impact exerted on the mitochondria involved a meticulous examination of the mitochondrial potential within MSC-derived glial-like cells, leveraging a nuanced morphological evaluation. It is widely acknowledged that a cell, when in an active state, exhibiting the hallmarks of normal physiological activity, boasts an elevated membrane potential. This foundational understanding sets the stage for the vivid presentation of the outcomes encapsulated in Figure 5A,B, capturing the nuances of the mitochondrial probe, elegantly depicted in a vibrant red hue, both pre and post the judicious application of the AOSP treatment. In a parallel narrative, Figure 5C,D delve into the results observed in the cellular samples, juxtaposing the states preceding and succeeding the administration of the AIP treatment. The discernible intensification in the red staining, as vividly portrayed in Figure 5B,D, unequivocally signifies the heightened mitochondrial membrane potential elicited in response to the administered therapeutic interventions. Identical outcomes were obtained through a comprehensive evaluation of the mitochondrial electron transport chain, utilizing the Enzyme-Linked Immunosorbent Assay (ELISA) technique. This detailed analysis, facilitated by the ELISA test, consistently corroborated the initial findings, providing robust and reproducible data on the functionality and efficiency of the electron transport chain within the mitochondria.

### 2.4. Safety Profile of the Treatment

The evaluation of treatment safety, particularly concerning the delicate nature of MSC-derived glial-like cells, required a thorough examination through a comprehensive hemolysis test, conducted in accordance with the rigorous standards outlined by ISO 10993 [20]. The clear findings obtained from this analysis substantiate and confirm that neither the AOSP (Figure 2A) nor the AIP (Figure 2B) induced any hemolytic processes, thus providing a definitive confirmation of the system’s inherent safety profile in MSC-derived glial-like cells. In an exhaustive investigation of the mutagenic potential inherent in the CMF treatment regimen, a comprehensive analysis was conducted through the Ames test, as shown in Table 1A,B.

### 2.5. In Vitro Mutagenic Profile Evaluation

**Figure 6:**
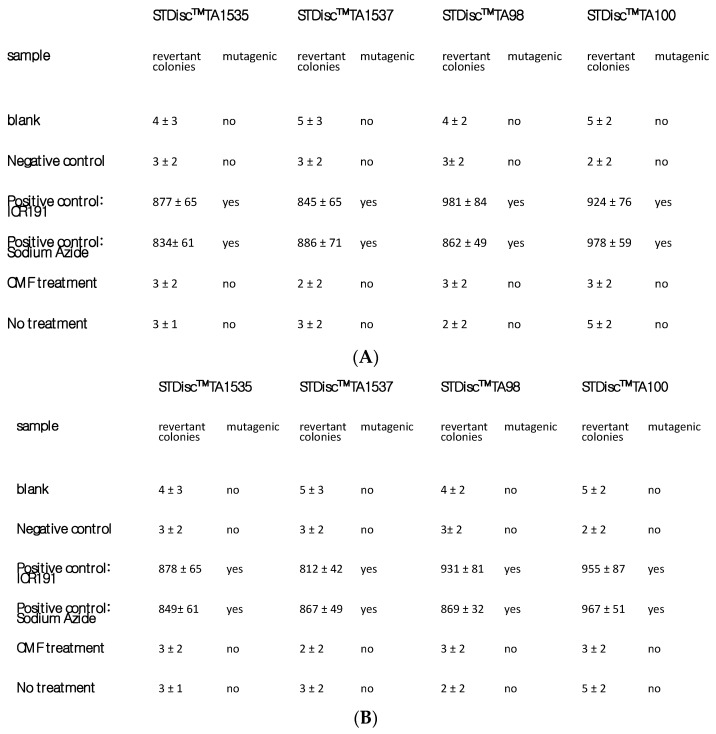
Ames test: (**A**) antioxidative stress program; and (**B**) anti-inflammatory program. ICR 191 is the Acridine mutagen.

In a meticulous exploration of the mutagenic potential inherent in the CMF treatment regimen, a comprehensive analysis was undertaken through the Ames test. The detailed findings, prominently showcased in Figure 6A,B, unequivocally establish the absence of any mutagenic events induced by either of the programs scrutinized. This robust and thorough examination further reinforces the safety and genetic stability of the treatment approach, instilling confidence in its potential application without triggering mutagenic concerns.

## 3. Discussion 

**Figure 7:**
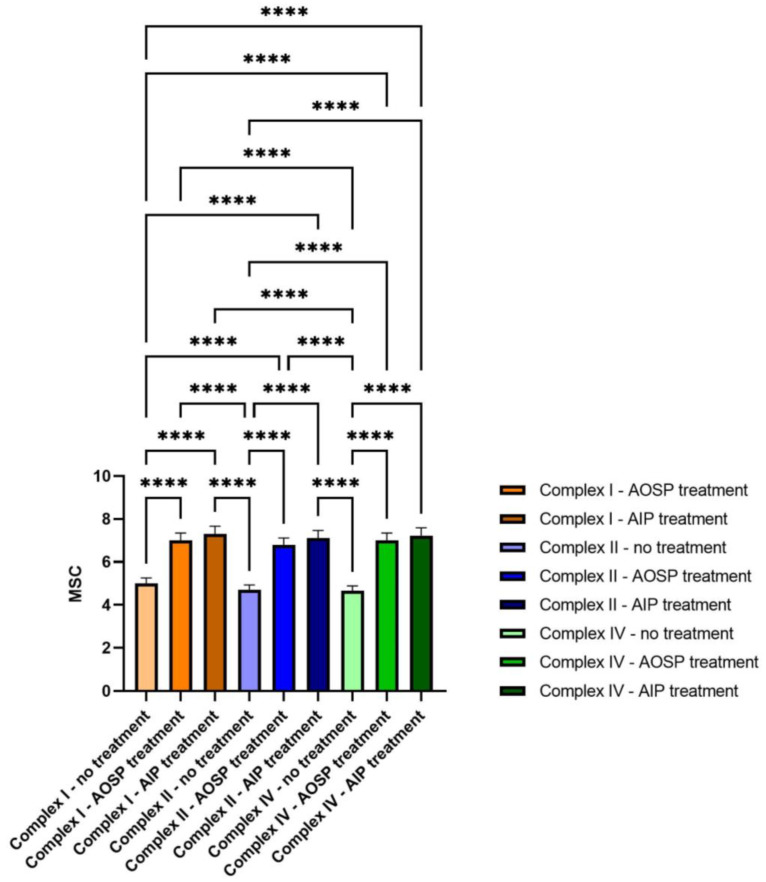
Mitochondrial electron transport chain (complex I, III, IV) detected with the ELISA Sampler Pack **** *p* value ≤ 0.05.

In the expansive field of physics-based biotechnology, pulsed electromagnetic fields (PEMFs) have emerged as a potent tool for health enhancement. A wealth of scientifically validated evidence, documented in numerous publications, confirms their biological safety and effectiveness in facilitating healing processes across diverse areas, including skin wounds, bone fractures, and issues related to joints and muscles. Recent years have seen an increased focus on their impact on the nervous system, suggesting their potential as an adjunctive therapy in neurodegenerative processes. In the context of tumor lines, PEMFs have demonstrated biological safety and efficacy by mitigating oxygen free radicals, which are typically involved in cell aging, degeneration, and decreased functionality. It is worth noting that the tumor-derived cell lines used in this study are distinct from those implicated in neurodegenerative diseases [21,22,23,24,25,26,27,28,29]. To conduct a preliminary in vitro analysis of the effects of complex magnetic fields (CMFs), mesenchymal stem cells (MSCs) differentiated into glial cells were selected as the target cells for this study. Two specific programs were analyzed: one designed to exert anti-inflammatory effects and the other targeting anti-ROS reactions. The initial assessments involved evaluating the cell viability rates correlated with mitochondrial function through an MTT assay. Undifferentiated MSCs and two tumor lines, representative of glial and neuronal cells, were included to ensure that the programs did not induce abnormal proliferative activity in tumor cells. Encouragingly, the results revealed no pathological alterations across all cell types, including those derived from MSC differentiation. The subsequent confirmation of these programs’ safety was achieved through Ames and hemocompatibility tests, aligning with the ISO 12000 guidelines [30]. The impact on ROS production, a central activity associated with CMFs, was validated on both MSCs and glial cells. The biological pathways activated by the programs were scrutinized through the study of miRNAs, primary biological entities controlling the activation or inhibition of various metabolic pathways. Notably, comprehensive studies on the miRNA effects of CMF are currently lacking [31,32]. The effects of the two programs on glial cells and MSCs were meticulously analyzed, as illustrated in Figure 6 and Figure 7, where heat maps vividly depict the overexpression or under-expression of key miRNAs. The pathways regulated by these miRNAs were thoroughly investigated, encompassing bone remodeling, DNA repair, neurotransmitter metabolism, cell–cell signaling, cell recognition, mitochondrial organization and biogenesis, lipid transport, cell cycle, and peptide metabolism. The AOSP demonstrated regulatory influence over lipid transportation, secretory activity, cellular organization, mitochondrial biogenesis, cell–cell signaling, cell growth, and vesicle-mediated transportation. Conversely, the AIP impacted plasma membrane organization and vesicle docking and reduced cell proliferation. These findings synergize with evidence from the MTT assay, suggesting that CMFs support glial cell growth by influencing plasma membrane organization, lipid production, and cell communication. The miRNAs predominantly involved in these processes fall into three families: 121, 127, and 142. These families are intricately linked to processes associated with extracellular matrix remodeling and tissue regeneration, offering promising prospects for treating neurodegenerative diseases. miRNAs 121, 127, and 142, by virtue of their involvement in these crucial processes, unveil new avenues for advancing the treatment landscape in neurodegenerative diseases. Our comprehensive study underscores the profound impact of complex magnetic fields (CMFs) on cellular physiology and heralds a promising frontier in therapeutic innovation [33,34,35,36,37]. By delving into the intricate mechanisms underpinning the CMF-mediated modulation of mitochondrial physiology, we unraveled a captivating narrative of potential therapeutic efficacy. These findings invite us to re-evaluate conventional paradigms in regenerative medicine and neurodegenerative disease therapy, offering a compelling rationale for integrating CMFs into our therapeutic arsenal. This opens new possibilities for mitigating neuropathic conditions and fostering tissue regeneration, suggesting that CMFs could be a revolutionary tool in future medical treatments.

## 4. Materials and Methods

### 4.1. CMF Treatment

The electronic device CMF Next (SX version; M.F.I. Medicina Fisica Integrata, Rome, Italy) emits pulsed multi-frequency electromagnetic fields between 1 and 250 μT. This CMFs generator is provided with different programs, each focused on the specific therapeutic effect that must be achieved. Each program comprises several different steps with variable intensity (1 to 250 µT), frequency (1 to 250 Hz), duration (1 to 4 min per step), and complex multi-frequency waveforms (enriched with harmonics).

In the present study, we tested the anti-inflammatory and anti-ROS programs. The tested cells were treated for up to five days, once a day.

### 4.2. Cell Cultures

Commercial cell lines, including mesenchymal stem cells (MSCs), the U251 MG human glioblastoma cell line, and mouse (B6/D2 F1 hybrid) catecholaminergic neuronal tumor cells, were seeded at a density of 5 × 104 cells/cm^2^. The cell cultures were maintained at 37 °C and 5% CO_2_, with the cell culture medium refreshed every 3 days. MSCs derived from diabetic patients were cultured in Dulbecco’s modified Eagle’s medium (DMEM, EuroClone) supplemented with 10% fetal bovine serum (FBS, EuroClone) or in specialized media for neuronal or glial differentiation. The exclusion criteria included a known or suspected cancer diagnosis, ongoing dialytic treatment for chronic renal failure, and a life expectancy of less than 1 year. Cell extraction followed previously published protocols for skin. Full-thickness skin biopsies measuring 3 cm × 1 cm were prepared, with the subcutaneous tissue removed and the biopsies cut into 1 mm × 1 mm pieces. These skin fragments underwent overnight digestion in DMEM and Worthington’s collagenase. After centrifugation and straining, the cell suspension was cultured in proliferating medium, consisting of DMEM-HAM’s F12, penicillin/streptomycin, EGF, FGF, and FBS. Neuronal and glial differentiation were induced using specialized media containing DMEM-F12, FBS, b27 serum-free supplement, NGF, antibiotics, forskolin, and heregulin β.

The cells were treated with the anti-inflammatory or anti-ROS program and analyzed for proliferation my means of an MTT test, cytotoxicity with a hemolytic test, mutagenesis activity with the AMES test, and ROS production and miRNA by sequencing the miRNA sequence (Figure 1).

### 4.3. MTT Assay

Three samples were prepared for each group. The MTT produced for each group was assessed after 5-day cell exposure. To determine the presence of viable cells, the MTT-based proliferation assay was performed according to the method described in [19]. In short, the samples were incubated for 3 h at 37 °C in 1 mL of 0.5 mg mL^−1^ MTT solution prepared in PBS. After the removal of the MTT solution by pipette, 0.5 mL of 10% DMSO in isopropanol was added to the samples at 37 °C for 30 min to extract formazan. Optical density (O.D.) values at 570 nm were recorded in duplicate on 200 μL aliquots deposited on microwell plates using a multilabel plate reader (Victor 3, Perkin Elmer, Milano, Italy).

### 4.4. Hemolysis Assay

The hemolysis assay was performed following standard practices, as established in ASTM F756, to evaluate the blood compatibility of the cells (1321N1 Cell Line) after the CMF treatment by either AOSP or AIP and without treatment [21,23]. The hemolysis assay indeed provides a valuable method for the preliminary screening of endosomolytic agents using naturally occurring biomembranes. However, it should be viewed as just one component among various tools available to drug delivery researchers for evaluating cytosolic delivery agents. The blood of three healthy New Zealand rabbits was pooled and diluted in phosphate-buffered saline (PBS; Lonza S.r.l., Milano, Italy) to achieve a total hemoglobin concentration of 10 ± 1 mg/mL. One mL of this blood was added to seven mL of the PBS extracts. Each sample was incubated for 3 h at 37 °C, then centrifuged for 15 min at 800× *g*. One mL of the resulting supernatant from all the samples was added to one mL of Drabkin’s reagent (Sigma-Aldrich, St. Louis, MO, USA) and incubated at room temperature for 15 min. The reaction product was quantified with a multilabel plate reader (Victor 3, Perkin Elmer, Milano, Italy) by measuring the optical density (OD) at 540 nm.

### 4.5. Ames Test

The mutagenic potential of the CMF treatment was evaluated with the Ames test using the Salmonella Mutagenicity Complete Test Kit (Moltox, Molecular toxicology Inc., Boone, NC, USA), as described previously [25]. The Ames test is indeed a frequently employed technique that employs bacteria to determine whether a specific chemical/physical treatment can induce mutations in the DNA of the test subject. This biological assay is formally employed to evaluate the mutagenic capacity of chemical compounds. In short, the content of the cells after treatment was extracted for (24 ± 2) h at (37 ± 1) °C using nutrient broth (blank) as the extraction vehicle. Four different strains of Salmonella were incubated for 48 h at 37 °C with the different extracts; then, the number of revertant colonies per plate was counted. Three replicates were performed per sample. If the number of reverted colonies was equivalent to that observed with the blank and negative control, the sample was considered to be non-mutagenic, whereas, if the number of reverted colonies was equivalent to that observed with the positive controls, the sample was considered mutagenic. In the graph are reported the strains of Salmonella tested.

### 4.6. Mitochondrial Membrane Potential

Cells were treated with MitoTracker Red CMXRos (Thermo Fisher Scientific, Waltham, MA, USA) for 30 min at 37 °C, following established protocols [27]. Following the washing steps, the cells were promptly visualized using a Confocal Microscope Eclipse Ti, 510/580 nm, outfitted with the Elements microscope imaging software V7, and a confocal laser scanning Olympus FV3000 microscope, both equipped with a 63× oil immersion objective (N.A. 1.4). Fluorescent puncta were manually counted to assess the signal intensity for each condition. Each condition was examined in five replicates, with four measurements taken per replicate. The ELISA Sampler Pack developed for analyzing the electron transport chain (specifically Complexes I, III, IV) by Assay Genie was employed with utmost precision, adhering strictly to a comprehensive protocol. This protocol encompassed the meticulous preparation and utilization of a calibration curve, which served as a critical component in the quantification of the enzymatic activities linked to the electron transport chain complexes. By following these rigorous procedures, we ensured methodological robustness and consistency, thereby obtaining precise and reliable results essential for advancing our understanding of mitochondrial function.

### 4.7. miRNA Extraction

Total RNA was isolated from the cells using the total RNA purification Plus kit (Norgen Biotek, Toronto, ON, Canada). The RNA quality and concentration of the samples were measured with the NanoDropTM ND-1000 (Thermo Fisher Scientific, Waltham, MA, USA). For one sample, 500 ng of total RNA was reverse-transcribed using an RT^2^ First Strand kit (Qiagen, Hilden, Germany) in a final reaction volume of 20 μL. Sequencing of all RNAs was carried out by Area Science Park (ASP, Trieste, Italy) with Illumina sequencing.

### 4.8. Library Preparation, RNA Sequencing, and Drop PCR

Libraries were prepared using 1 μg of total RNA with the TruSeq Sample Preparation RNA Kit (Illumina, Inc., San Diego, CA, USA), following the manufacturer’s protocol without modifications [29]. The quality of cDNA libraries was assessed using the DNA 1000 Chip on the Bioanalyzer 2100 (Agilent Technologies, Santa Clara, CA, USA), and quantification was performed using the QubitTM dsDNA BR Assay Kit on the Qubit 2.0 Fluorometer (Thermo Fisher Scientific, Waltham, MA, USA). Sequencing was conducted on a Novaseq 6000 sequencer (Illumina, Inc., San Diego, CA, USA), generating approximately 25 million 2 × 150 bp paired-end reads per sample. Demultiplexing and FASTQ file generation were performed using the Illumina BCL2FASTQ v2.20 software. The resulting reads were aligned to the complete human genome using the Spliced Transcripts Alignment to a Reference (STAR) algorithm (version 2.7.3) with hg38 Genome Assembly and Gencode.v35 as the gene definition. The mapped reads were processed using the feature counts function of the Rsubread package, and the gene counts obtained were used for differential expression analysis with the Deseq2 package. Genes showing significant differential expression (|log_2_(FC)|≤ or ≥1 and corrected *p*-value ≤ 0.05) were subjected to a pathway enrichment analysis using the IPA system (30/11/2003 Systems, www.ingenuity.com, Toronto, ON, Canada).

Total RNA was extracted from the cells and tissues using the miRNeasy Mini Kit (Qiagen, Milan, Italy) and QIAamp RNeasy FFPE Kit (Qiagen, Milan, Italy), respectively. The RNA samples were quantified spectrophotometrically with the NanoDrop 2000 (Thermo, Milan, Italy) and stored at −80 °C until further analysis. The miRNAs were reverse-transcribed using the miRCURY LNA RT kit (Qiagen, Milan, Italy), while total mRNA was reverse-transcribed using ImProm-II™ Reverse Transcriptase. The resultant products were stored at −20 °C until required for analysis. The quantification of hsa-miR expressions was performed using droplet digital PCR (ddPCR) with the QX200 ddPCR system (Bio-Rad, Segrate, Italy). ddPCR enables the absolute quantification of nucleic acid molecules in biological and clinical samples, obviating the need for normalization with housekeeping genes. The ddPCR Supermix for Probes (no dUTP) (Bio-Rad, Segrate, Italy) was combined with the TaqMan™ Gene Expression Assay (FAM) for HOXA13 (Thermo, Milan, Italy; Assay ID: Hs04194761_s1). Following mix preparation, an emulsion was generated using an automated droplet generator (Bio-Rad, Segrate, Italy). The ddPCR plate was heat-sealed and cycled in the SimpliAmp Thermal Cycler (Thermo Fisher Scientific, Milan, Italy), with plate reading conducted using the QX 200 Droplet Reader (Bio-Rad, Segrate, Italy). For normalization, SNORD44 and B2M were used as housekeeping genes for hsa-miR-1249-3p and HOXA13 mRNA, respectively. The expression levels were reported as copies per microliter (copies/μL) of hsa-miR-1249-3p and HOXA13, normalized to the SNORD44 and B2M housekeeping genes, respectively. The amplification conditions were as follows: (i) for hsa-miR-1249-3p and SNORD44, initial enzyme activation at 95 °C for 5 min, followed by 40 cycles of 30 s at 94 °C, 1 min at 56 °C, with final steps of 5 min at 4 °C and 5 min at 90 °C; and (ii) for HOXA13 and B2M, initial enzyme activation at 95 °C for 10 min, followed by 40 cycles of 30 s at 94 °C, 1 min at 55 °C, and 10 min at 98 °C. Where applicable, the results are presented as mean levels of miRNA and/or mRNA ± standard deviation (SD) or the standard error of the mean (SEM).

### 4.9. Statistics

A one-way analysis of variance was used for the data analyses. In addition, *t*-tests were used to ascertain significant differences (*p* < 0.05). Repeatability was calculated as the standard deviation of the difference between measurements. Any testing was performed in the SPSS 16.0 software (SPSS Inc., Chicago, IL, USA; license of the University of Ferrara, Ferrara, Italy).

## 5. Conclusions

In essence, our research illuminates previously obscured pathways, shedding light on novel avenues for therapeutic intervention. By elucidating the interplay between CMFs and mitochondrial dynamics, we unlocked a treasure trove of possibilities for mitigating neuropathic conditions and fostering tissue regeneration. This paradigm shift challenges us to reimagine the treatment landscape, transcending traditional boundaries to embrace the transformative potential of CMFs. One aspect of CMF therapy that warrants attention is its immunomodulatory effect, which plays a crucial role in tissue healing and regeneration. Additionally, CMF therapy has been shown to mitigate oxidative stress by combating ROS, thereby promoting a favorable microenvironment for tissue repair. CMF therapy has been demonstrated to modulate various components of the immune system, including immune cell proliferation, cytokine production, and inflammatory responses. Oxidative stress, characterized by an imbalance between ROS production and antioxidant defenses, can impair tissue healing and regeneration. CMF therapy has been shown to exert antioxidative effects by reducing the ROS levels and enhancing antioxidant enzyme activity. By modulating oxidative stress, CMF therapy creates a more favorable environment for cellular proliferation, differentiation, and tissue remodeling, ultimately facilitating the regenerative process. As we embark on this journey of discovery, our study serves as a guiding beacon, navigating the uncharted waters of regenerative medicine and neurodegenerative disease therapy. With each revelation, we inch closer to realizing the full potential of CMFs as a cornerstone in our quest for healing and restoration. Let us seize this opportunity to redefine the boundaries of possibility, harnessing the power of CMFs to chart a course towards a brighter and more resilient future.

## Figures and Tables

**Table 1:** Hemolysis assay: (**A**) antioxidative stress program; and (**B**) anti-inflammatory program.

(A)			
**Scheme**	**OD**	**Hemolysis index**	**Results**
Positive control	0.943% ± 0.023	100%	Hemolytic
Negative Control	0.023% ± 0.067	0	Non hemolytic
CMF treatment	0.034% ± 0.056	0.042%	Non hemolytic
No Treatment	0.033% ± 0.072	0.038%	Non hemolytic
(B)			
**Sample**	**OD**	**Hemolysis index**	**Results**
Positive control	0.875% ± 0.018	100%	Hemolytic
Negative Control	0.013% ± 0.054	0	Non hemolytic
CMF treatment	0.023% ± 0.042	0.087%	Non hemolytic
No Treatment	0.029% ± 0.042	0.096%	Non hemolytic

## Data Availability

The data presented in this study are available in the article.
